# Evaluation of the influence of tumor location and size on the difference of dose calculation between Ray Tracing algorithm and Fast Monte Carlo algorithm in stereotactic body radiotherapy of non‐small cell lung cancer using CyberKnife

**DOI:** 10.1120/jacmp.v14i5.4280

**Published:** 2013-09-06

**Authors:** Vincent W.C. Wu, Kwok‐wah Tam, Shun‐ming Tong

**Affiliations:** ^1^ Department of Health Technology & Informatics Hong Kong Polytechnic University Hung Hom Hong Kong SAR; ^2^ Teresa Po CyberKnife Center Hong Kong Adventist Hospital Wan Chai Hong Kong SAR

**Keywords:** Ray Tracing algorithm, Fast Monte Carlo algorithm, CyberKnife, lung cancer, stereotactic body radiotherapy, dose calculation

## Abstract

This study evaluated the extent of improvement in dose predication accuracy achieved by the Fast Monte Carlo algorithm (MC) compared to the Ray Tracing algorithm (RAT) in stereotactic body radiotherapy (SBRT) of non‐small cell lung cancer (NSCLC), and how their differences were influenced by the tumor site and size. Thirty‐three NSCLC patients treated with SBRT by CyberKnife in 2011 were recruited. They were divided into the central target group (n=17) and peripheral target group (n=16) according to the RTOG 0236 guidelines. Each group was further divided into the large and small target subgroups. After the computation of treatment plans using RAT, a MC plan was generated using the same patient data and treatment parameters. Apart from the target reference point dose measurements, various dose parameters for the planning target volume (PTV) and organs at risk (OARs) were assessed. In addition, the “Fractional Deviation” (FDev) was also calculated for comparison, which was defined as the ratio of the RAT and MC values. For peripheral lung cases, RAT produced significantly higher dose values in all the reference points than MC. The FDev of all reference point doses and dose parameters was greater in the small target than the large target subgroup. For central lung cases, there was no significant reference point and OAR dose differences between RAT and MC. When comparing between the small target and large target subgroups, the FDev values of all the dose parameters and reference point doses did not show significant difference. Despite the shorter computation time, RAT was inferior to MC, in which the target dose was usually overestimated. RAT would not be recommended for SBRT of peripheral lung tumors regardless of the target size. However, it could be considered for large central lung tumors because its performance was comparable to MC.

PACS number: 87

## I. INTRODUCTION

Lung cancer is one of the most prevalent malignancies worldwide and has the highest mortality. Early stage non‐small cell carcinoma of lung (NSCLC) is often treated by stererotactic body radiotherapy (SBRT) using CyberKnife, from which satisfactory results have been reported.^1^, ^2^, ^3^, ^4^, ^5^ SBRT is the use of stereotactic radiotherapy outside the cranium, in which multiple radiation beams are directed at a relatively small target with high precision. CyberKnife (Accuray Inc., Sunnyvale, CA) is a frameless image‐guided radiosurgical system that delivers radiation treatment using a robot‐mounted 6 MV compact linear accelerator. The highly flexible nonisocentric beam delivery facilitates the implementation of stereotactic body radiotherapy (SBRT), which is a highly conformal treatment with steep dose gradients at the target‐normal tissue boundary.^(6)^

Since SBRT delivers relatively high dose per fraction in small number of fractions, accurate dose prediction in the treatment region during treatment planning is essential to understanding the dose levels to the target volumes and normal organs, and it is generated by the dose calculation algorithms in the radiotherapy treatment planning system (TPS). Currently, the overall dosimetric accuracy in radiotherapy recommended by AAPM is 5%, in which the dose calculation should be kept within 3%.^(7)^ The situation becomes more challenging when the treatment site contains complex heterogeneous tissue densities, in which the dose calculation algorithm has to correct for the effects of transient electronic disequilibrium between the tissue interfaces.^(8)^ Lung tumor is a soft tissue tumor surrounded by lung tissue, thoracic bone cage (ribs and spine), and the mediastinal soft tissues. These tissues have very different densities and can be a challenge in accurate dose calculation at the different tissue interfaces, especially for the small radiation fields used in CyberKnife.^(9)^

The Ray Tracing (RAT) algorithm has been used by the Cyberknife Multiplan TPS for the planning of CyberKnife treatments. RAT belongs to the correction‐based algorithm, in which the off‐center ratio, tissue‐phantom ratio, and collimator output factor that are measured under reference conditions, are corrected for the patient's geometry. The absorbed dose is calculated by assuming the effective depth as determined by the density variation along the beam path. RAT does not take into account effects arising due to the variation of tissue heterogeneity and electronic disequilibrium at tissue interface, and therefore is regarded as a less accurate algorithm.

Recently, the Fast Monte Carlo (MC) algorithm, which is a modification of the full Monte Carlo simulation, has been introduced in the CyberKnife Multiplan. MC algorithm predicts the absorbed dose by simulating the electron and photons transport based on a probability distribution derived from the first principles. MC algorithm takes into account the electronic disequilibrium, and is generally accepted as the most accurate algorithm at present. However, the main trade‐off is its relatively long processing time. Recently, MC algorithm has been introduced in the CyberKnife TPS with a range of “uncertainty levels” from 0.1% to 4%, in which the higher uncertainty level is associated with less number of photon simulation histories, and therefore is less accurate. Our study aimed to evaluate how much improvement in dose predication accuracy could be achieved by MC algorithm when compared to the RAT algorithm in the SBRT treatment of NSCLC, and how their differences were influenced by the tumor site and size.

## II. MATERIALS AND METHODS

This is a retrospective study on 33 stage I and II NSCLC patients treated with SBRT by CyberKnife in 2011. They were divided into the central target group (n=17) and peripheral target groups (n=16) with reference to the descriptions from trial of the Lung Cancer Stereotactic Radiotherapy versus Surgery (STARS) in RTOG‐O236.^(10)^ The central lesion was defined as tumor within 2 cm from the mediastinum, pulmonary, and vertebral structures, whereas tumors arising from the rest of the lung were classified as peripheral lesions. In addition, in order to study the effect of tumor size on the dosimetric outcome, each group was further divided into the large and small target subgroups according to the criterion suggested by van der Voort van Zyp et al.,^(11)^ in which the large target subgroup referred to planning target volume (PTV) of over 27 ${\text{cm}}^{3}$, while the small target subgroup referred to PTV of smaller than $27\;{\text{cm}}^{3}$.

The original treatment plan (RAT Plan) of each patient was computed with the CT taken when the patient was in normal breathing condition. The CT images (1.5 mm thick) were loaded into the CyberKnife Multiplan (Version 4.1) TPS, in which the PTV (by adding 5 mm to the clinical target volume) and organs at risk (OARs) including the spinal cord, oesophagus, and lung were contoured. The treatment plan followed the protocol of the local department in which a tumor dose of 50 Gy in 3 to 5 fractions was prescribed to the PTV at the 80% isodose level. The prescribed dose should at least cover 95% volume of the PTV, and the dose coverage of PTV should fall between ‐10% and +25% of the prescribed dose. All treatments were delivered using cone collimation. No avoidance zone was applied and the total number of beams (nodes) for each patient was between 112 and 195. After setting the dose constraints to the targets and OARs, a treatment plan was generated through the sequential optimization process. Isodose display was obtained after dose calculation using the RAT algorithm. The computation grid used for calculation was $1.5\;{\text{mm}}^{5}$. Using the same patient data, same beam number, directions, weights, and monitor units, another treatment plan (MC Plan) was generated using the same TPS with the MC algorithm set at 1% uncertainty level. The reason for using 1% uncertainty level is it was relatively accurate and with a reasonable operation time. The Fast Monte Carlo algorithm improved the speed of computation, and it was reported to be within ± 0.5% relative to the MC calculation in hetereogeneous conditions using phantom by Ma et al.^(12)^

**Figure 1:**
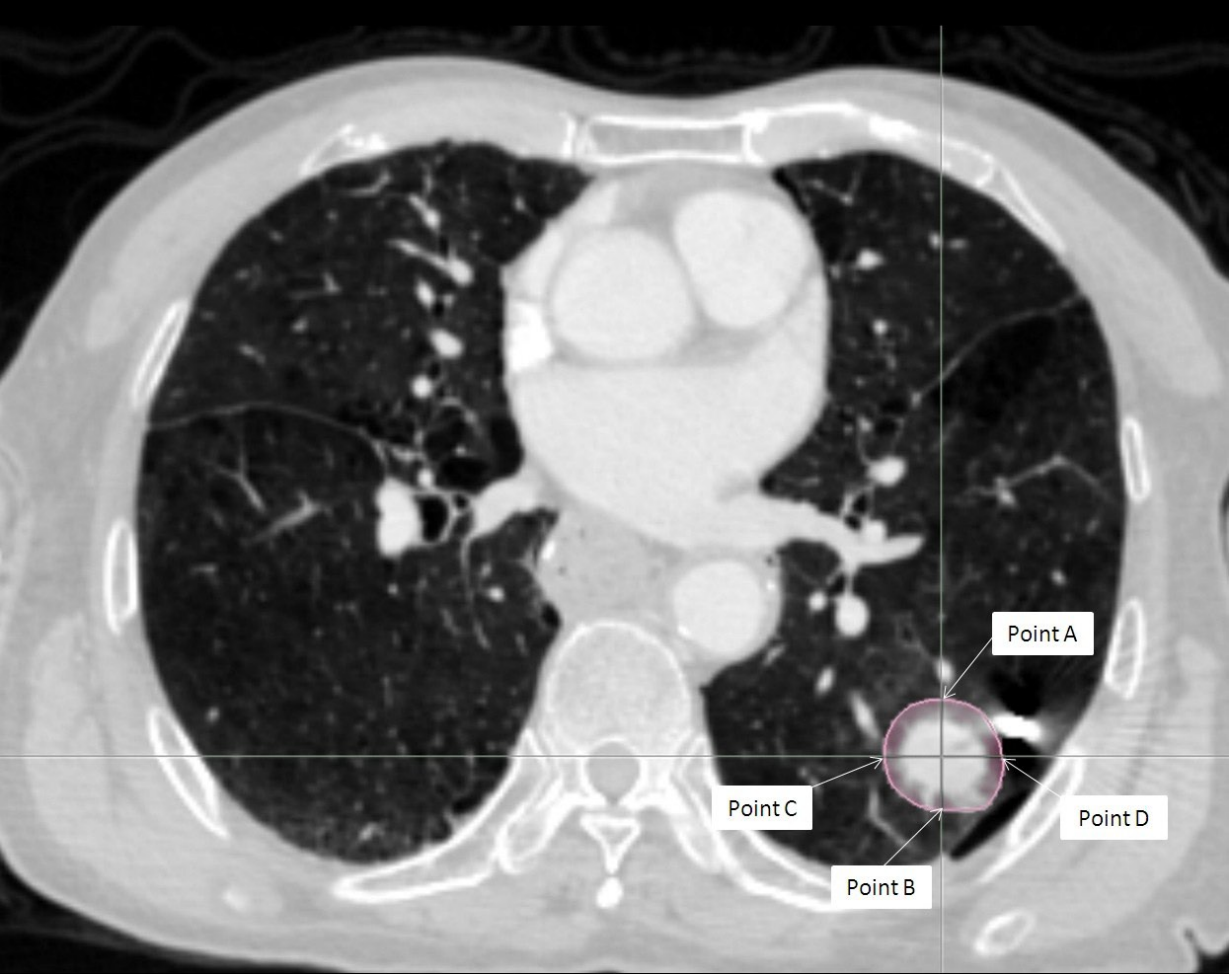
A CT slice at the center of the planning target volume (PTV) showing the locations of the four reference points for a peripheral lung cancer case. Points A, B, and C were situated at the anterior, posterior, and medial borders of PTV, respectively, which were near the soft tissue and lung interface. Point D was at the lateral border, which was in between soft tissues or close to the rib bone.

**Figure 2:**
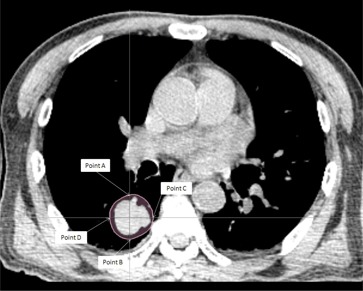
A CT slice at the center of the planning target volume (PTV) showing the locations of the four reference points for a central lung cancer case. Points A, B, and C were situated at the anterior, posterior, and medial borders of PTV, respectively, which were in between soft tissues or close to the bone. Point D was at the lateral border, which was in between soft tissues and lung tissue.

Reference point doses were used to analyze the dosimetric information. These reference points were defined at the anterior, posterior, lateral, and medial boundaries of the PTV in each corresponding slice (Figs. 1 and 2). These points were situated at or close to the tumor‐lung or tumor‐soft tissue interface and sites of steep dose gradient, which were most challenging to the dose calculation algorithms, and at the same time affected the dosimetric prediction of the target. The total number of reference points for each patient was between 48 and 60. Apart from the reference point dose measurements, other dosimetric parameters of the target and OARs (ipsilateral lung, oesophagus, and spinal cord) were also compared. The PTV coverage was assessed by measuring the $D_{2},D_{98}$, conformity index (CI), and homogeneity index (HI), whereas for the OARs, $V_{20},V_{30}$, and mean dose were used for the ipsilateral lung, and the maximum, mean, and $D_{2}$ doses were used for the oesophagus and spinal cord. The calculation of CI was adopted from the following formula:^(13)^

(1) $$\text{CI}=\text{target volume}\times \text{prescribed isodose vol}./{\left(\text{vol}.\;\text{of target covered by prescribed isodose}\right)}^{2}$$

The closer the value of CI to 1.0, the better would be the target dose conformity. The HI was calculated by:^(14)^

(2) $$(D_{2}-D_{98})/D_{50}$$

in which $D_{2},D_{98}$, and $D_{50}$ were the doses that covered 2%, 98%, and 50% of the target volume, respectively. The larger the value of HI where the difference between the high and low doses was great, the more heterogeneous would be the target dose. The reference acceptable values for the treatment plans were: CI < 1.50; HI< 0.50; $D_{2}\;<\;62.5\;\text{Gy}$; $D_{98}\;>\;40\;\text{Gy}$, and $D_{95}$ fell between 45 Gy and 55 Gy.

The mean reference point doses in the plans generated from the RAT and MC algorithms were calculated and compared. Paired *t*‐test was used to test the significance of their differences. In addition to the dose parameters, the “Fractional Deviation” (FDev) was also calculated for the dosimetric parameters. FDev was defined as the ratio of the value of the dosimetric parameter between the RAT and MC (i.e., FDev = RAT/MC). For example, if both algorithms presented with the same dosimetric value, a value of 1.0 would be obtained which implied that there was perfect match between the two algorithms. To evaluate the differences between peripheral and central lung tumors, the mean FDev of the RAT algorithm was calculated and compared with that of the MC algorithm using *t*‐test. In addition to studying the effect of PTV size under each tumor site group, the FDev of the dose parameters were compared between the small target and large target subgroups. Student's *t*‐test or Mann‐Whitney U test (depending on the normality of the data) was used to test the significance of their differences. The hypothesis for the paired *t*‐test or Mann‐Whitney U test was that the values of the dosimetric parameters obtained from MC were significantly different from those of the RAT. In addition, the calculation time was recorded using a stopwatch, and the mean calculation time was compared between the two algorithms. Paired *t*‐test was used to test the significance of the difference.

## III. RESULTS

There were 12 and five cases belonging to the small target and large target subgroup, respectively, in the peripheral lung cases, and eight cases in both subgroups in the central lung cases. With regard to the reference point doses of in all the 33 cases, the RAT algorithm produced dose values of 10.9%‐13.1% higher than that of the MC algorithm, with all the differences reached statistical significance (p < 0.05) (Table 1). The RAT algorithm also produced significantly higher PTV doses, but lower CI and HI. In addition, the mean calculation time for MC algorithm was 44.6 ± 31.9 minutes, which was significantly longer than that of the RAT algorithm (4.8 ± 2.8 minutes, p < 0.001).

**Table 1:** Dosimetric comparison between Ray Tracing and Fast Monte Carlo algorithms at the reference points in the SBRT of all lung cancer cases (n=33) using CyberKnife

	*RAT* Mean ± SD	*MC* Mean ± SD	*Paired t*‐test *p‐value*
Reference Points			
Point A (Gy)	50.2±10.4	43.63±9.0	0.007
Point B (Gy)	51.5±10.8	45.89±10.3	0.034
Point C (Gy)	50.5±10.9	44.78±9.4	0.027
Point D (Gy)	52.3±11.3	45.58±10.8	0.016
PTV			
CI	1.22±0.05	1.39±0.38	0.013
HI	0.25±0.05	0.34±0.08	0.001
$D_{2}\;(\text{Gy})$	62.5±10.2	58.5±9.3	0.101
$D_{95}\;(\text{Gy})$	50.0±9.0	42.9±6.9	0.001
$D_{98}\;(\text{Gy})$	47.8±8.8	40.5±6.4	0.001

RAT = Ray Tracing algorithm; MC = Fast Monte Carlo algorithm.

### A. Peripheral lung cases

**Figure 3:**
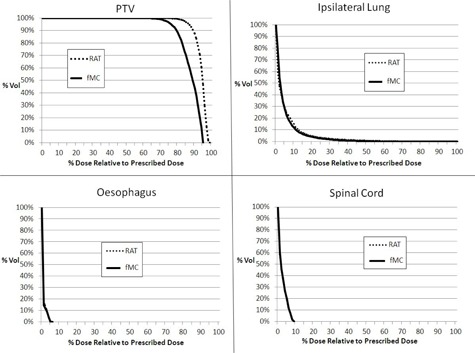
Comparison of the average dose‐volume histograms (DVHs) of the PTV and OARs between RAT and MC algorithms for the CyberKnife plans of the peripheral lung tumors.

The RAT algorithm produced dose values of about 15% higher in all the reference points when compared with the MC algorithm, with the differences of Point A and C reaching significance (p = 0.039 and 0.041, respectively) (Table 2). For the PTV, MC algorithm produced significantly larger CI and HI values when compared to that of the RAT algorithm. The RAT algorithm demonstrated greater values in the other dose parameters, with the $D_{95}$ and $D_{98}$ reaching statistical significance (p = 0.015 and 0.011, respectively). There was no significant difference in the ipsilateral lung dose between the two algorithms, although the values of RAT algorithm were slightly higher. The average DVHs of the PTV and OARs are shown in Fig. 3. When comparing between the small target and large target subgroups, the FDev values of all the reference point doses and dose parameters were greater in the small target subgroup, in which the differences of Point D dose, CI, $D_{95}$, and $D_{98}$ were significant.

**Table 2:** Dosimetric comparison between Ray Tracing and Fast Monte Carlo algorithms in the SBRT of peripheral lung cancer cases using CyberKnife

	*Dose Parameters*			*Fractional Deviation (RAT/MC)*		
	*All Cases* (n=17)			*Small Target* (n=12)	*Large Target* (n=5)	
	*RAT* Mean ± SD	*MC* Mean ± SD	*t‐test p‐value*	*Median*	*Median*	*MWU test*
Ref Points						
Point A (Gy)	52.1±11.2	44.4±9.6	0.039	1.20	1.12	0.226
Point B (Gy)	53.8±11.4	46.4±10.9	0.062	1.17	1.12	0.430
Point C (Gy)	52.7±13.1	43.9±10.9	0.041	1.24	1.11	0.061
Point D (Gy)	53.8±12.5	45.8±11.6	0.061	1.23	1.05	0.012
PTV						
CI	1.21±0.06	1.50±0.41	0.007	0.82	0.96	0.008
HI	0.24±0.04	0.34±0.09	0.001	0.73	0.81	0.064
$D_{2}\;(\text{Gy})$	63.0±12.9	57.0±11.8	0.167	1.14	1.04	0.113
$D_{95}\;(\text{Gy})$	51.0±10.6	41.7±9.6	0.015	1.29	1.12	0.022
$D_{98}\;(\text{Gy})$	48.0±10.3	39.5±9.0	0.011	1.30	1.13	0.001
Ip. Lung						
$V_{20}\;(\%)$	5.3±4.8	4.8±4.6	0.769	1.10	1.00	0.712
$V_{30}\;(\%)$	2.7±2.5	2.0±2.2	0.405	1.07	1.03	0.883
$D_{\text{mean}}\;(\text{Gy})$	4.6±3.0	4.4±3.0	0.854	1.05	1.04	0.970

RAT = Ray Tracing algorithm; MC = Fast Monte Carlo algorithm; MWU = Mann‐Whitney U test; PTV = planning target volume; CI = Conformity Index; HI = Homogeneity Index; Ip. = Ipsilateral, D = mean dose.

### B. Central lung cases

**Figure 4:**
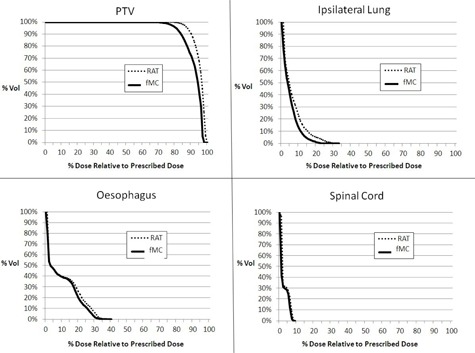
Comparison of the average dose‐volume histograms (DVHs) of the PTV and OARs between RAT and MC algorithms for the CyberKnife plans of the central lung tumors.

Despite the fact that the RAT algorithm showed higher reference point doses than MC, none of them showed significant difference (Table 3). The MC algorithm showed significantly greater CI and HI values than the RAT algorithm. The rest of the PTV parameters did not show significant differences. For the OAR dose parameters, the RAT algorithm showed relatively greater values than MC. However, only the $D_{\text{mean}}$ of the ipsilateral lung showed marginal significance. The average DVHs of the PTV and OARs are shown in Fig. 4. When comparing between the small target and large target subgroups, the FDev values of all the dose parameters and reference point doses did not showed any significant differences, despite the fact that the majority of the deviations in the small target subgroup were greater.

**Table 3:** Dosimetric comparison between Ray Tracing and Fast Monte Carlo algorithms in the SBRT of central lung cancer cases using CyberKnife

	*Dose Parameters*			*Fractional Deviation (RAT/MC)*		
	*All Cases* (n=16)			*Small Target* (n=8)	*Large Target* (n=8)	
	*RAT* Mean ± SD	*MC* Mean ± SD	*t‐test p‐value*	*Median* ±SD	*Median* ±SD	*t‐test p‐value*
Ref Points						
Point A (Gy)	48.3±9.5	42.9±8.4	0.098	1.13±0.22	1.12±0.21	0.927
Point B (Gy)	49.2±10.2	45.4±9.7	0.281	1.09±0.23	1.08±0.23	0.932
Point C (Gy)	48.2±8.6	45.6±8.0	0.386	1.08±0.18	1.01±0.17	0.437
Point D (Gy)	50.8±10.1	45.4±10.0	0.136	1.14±0.20	1.07±0.23	0.527
PTV						
CI	1.27±0.08	1.46±0.23	0.004	0.90±0.05	0.87±0.11	0.494
HI	0.25±0.05	0.33±0.06	0.001	0.76±0.02	0.77±0.03	0.446
$D_{2}\;(\text{Gy})$	57.3±11.4	54.8±10.5	0.530	1.06±0.33	1.01±0.19	0.716
$D_{95}\;(\text{Gy})$	46.1±8.4	40.9±7.8	0.076	1.15±0.20	1.09±0.23	0.587
$D_{98}\;(\text{Gy})$	43.8±8.3	38.5±7.4	0.067	1.16±0.25	1.09±0.21	0.554
Ip. Lung						
$V_{20}\;(\%)$	6.4±5.0	5.6±4.7	0.642	1.23±0.70	1.12±0.81	0.776
$V_{30}\;(\%)$	2.8±2.0	2.1±1.7	0.285	1.31±0.72	1.45±0.85	0.728
$D_{\text{mean}}\;(\text{Gy})$	6.7±3.2	6.3±3.2	0.047	1.05±0.49	1.06±0.51	0.969
Oeso.						
$D_{\text{max}}\;(\text{Gy})$	16.3±11.3	16.1±10.9	0.946	1.04±0.82	0.99±0.63	0.894
$D_{2}\;(\text{Gy})$	16.4±9.2	16.2±8.9	0.931	1.01±0.56	1.05±0.53	0.886
$D_{\text{mean}}\;(\text{Gy})$	3.8±2.6	3.7±2.5	0.886	1.00±0.92	1.03±0.83	0.947
Sp. Cord						
$D_{\text{max}}\;(\text{Gy})$	12.2±7.9	11.8±7.7	0.895	1.04±0.72	1.03±0.75	0.979
$D_{2}\;(\text{Gy})$	13.1±6.9	12.6±6.8	0.825	1.05±0.55	1.03±0.58	0.953
$D_{\text{mean}}\;(\text{Gy})$	2.7±1.7	2.3±1.6	0.552	1.15±0.74	1.20±0.85	0.902

RAT = Ray Tracing algorithm; MC = Fast Monte Carlo algorithm; MWU = Mann‐Whitney U test; PTV = planning target volume; CI = Conformity Index; HI = Homogeneity Index; Ip. = Ipsilateral; Dm = mean dose.

### C. Peripheral lung vs. central lung cases

For the reference points, the ranges of FDev were 1.18–1.21 and 1.06–1.13 for the peripheral lung and central lung, respectively (Table 4). The peripheral lung showed greater FDev values than the central lung in all reference point doses, in which the differences of Point B and C reached significance (p = 0.017 and 0.001, respectively). For the PTV dose parameters, the peripheral lung demonstrated slightly greater FDev values than that of the central lung, with only $D_{2}$ showing significant difference (p = 0.026).

**Table 4:** Comparison of RAT‐MC fractional deviations (RAT/MC) in reference point and target doses between the SBRT of peripheral lung and central lung using CyberKnife

	*Fractional Deviation (RAT/MC)*		
	*Peripheral Lung* (n=17)	*Central Lung* (n=16)	*t‐test*
	Mean ± SD	Mean ± SD	*p‐value*
Reference Points			
Point A (Gy)	1.18±0.17	1.13±0.11	0.327
Point B (Gy)	1.18±0.13	1.09±0.06	0.017
Point C (Gy)	1.21±0.12	1.06±0.06	0.001
Point D (Gy)	1.20±0.22	1.13±0.15	0.297
PTV			
CI	0.86±0.19	0.89±0.14	0.611
HI	0.76±0.18	0.77±0.14	0.733
$D_{2}\;(\text{Gy})$	1.11±0.09	1.05±0.05	0.026
$D_{95}\;(\text{Gy})$	1.24±0.20	1.13±0.08	0.461
$D_{98}\;(\text{Gy})$	1.25±0.23	1.14±0.10	0.521

RAT = Ray Tracing algorithm; MC = Fast Monte Carlo algorithm; PTV = planning target volume; CI = Conformity Index; HI = Homogeneity Index.

## IV. DISCUSSION

Our study compared the MC algorithm with the RAT algorithm in the CyberKnife SBRT of NSCLC. Taking the MC algorithm as the gold standard, the differences in the calculated dose distribution between the two algorithms revealed the performance of the RAT algorithm.

In the overall comparison, the RAT algorithm was found to have overestimated the doses by 10.9%‐13.1% at the reference points, which were the interfaces between the target and surrounding tissues. This implied that after accounting for density heterogeneity, the actual doses delivered to the patient using the RAT algorithms might range from 38.2–45.9 Gy (in 3 to 5 fractions). The finding was consistent with the finding by Sharma et al.^(15)^ Since lung tumor was mainly surrounded by the low‐density lung tissue, radiation beams usually traversed a considerable distance of low‐density tissue before reaching the target; this would reduce the dose to the target due to the “build down” effect of photon beam.^(7)^ Therefore, failure to correct this effect would lead to overestimation of the dose. Such difference found in this study illustrated the extent of inadequacy of the RAT algorithm in the modeling of secondary electronic disequilibrium at the tumor‐lung tissue interface.^(16)^

## A. Peripheral lung cases

An average of about 15% overestimation of the reference point doses was observed in the RAT algorithm. This indicated that the RAT algorithm was less capable of handling such anatomical condition. The situation was worse in Points A and C, because most of the radiation beams were directed from the anterior directions in SBRT of lung cases; the beams would have travelled a longer path of low‐density lung tissue before reaching these points. This posed a problem to the RAT algorithm. Similarly, this was also the main cause leading to the relative large differences of the PTV dose between the two algorithms. It should be noted that using the MC algorithm, the target conformity and homogeneity were deteriorated from the original plan due to a more accurate modeling of secondary electronic disequilibrium at the tumor‐lung tissue interface leading to a broadened beam penumbra.^(9)^ However, since such effect was not present in the ipslateral lung, no significant difference was seen between the two algorithms in this OAR.

Comparing the two target size subgroups, smaller targets demonstrated greater dose differences (FDev values) in the PTV dose parameters between the two algorithms. This echoed the report from Haedinger et al.,^(16)^ which stated that small targets might lead to insufficient dose to the target using the correction‐based algorithm.

### B. Central lung cases

Although similar patterns of dosimetric outcome were observed in the central lung cases as in the peripheral lung cases, the differences between the two algorithms were less obvious. Furthermore, the effect of target size was also not significant, as none of the FDev values demonstrated significant differences. Since the central tumor was commonly surrounded by soft tissues, the chance for FDev values to be subjected to extreme tissue density changes, such as in the tumor‐lung tissue interface, would be lower. As a result, the magnitude of secondary electronic disequilibrium was smaller. Since such condition was less difficult for the RAT algorithm, the gap between the two algorithms was smaller.

### C. Peripheral lung vs. central lung cases

In the peripheral lung cases, many of the radiation beams had to pass through a larger lung volume before reaching the target and a greater discrepancy in the calculated dose by the RAT algorithm that the central lung target would be expected. This was reflected in the greater FDev values of the peripheral lung group. Since in the central lung cases, Points B and C were surrounded by less volume of lung tissues than in the case of the peripheral lung cases (Figs. 1 and 2), therefore more obvious differences in FDev values between the two groups were observed. For the same reason, the FDev values of the PTV dose parameters for the peripheral lung were also greater than that of the central lung cases, though only the difference of the $D_{2}$ value was statistically significant. Our result was in line with another similar study by van der Voort van Zyp et al.^(11)^ on NSCLC, but comparing equivalent path‐length algorithm with MC in linear accelerator. They reported that there was an increased in dose inhomogeneity in peripheral tumors when compared with central tumors, and recommended a different prescription for different tumor size.

We assumed that the MC Algorithm used with 1% uncertainty level was the gold standard in radiotherapy dose calculation. In the treatment planning of CyberKnife, if the RAT algorithm could produce comparable dosimetric outcome as the MC algorithm, it would be welcome by the department, as its expected shorter computation time would bring about better economical value. In our study, the RAT algorithm was in general inferior to the MC algorithm, in which the target dose was usually overestimated. This implied that the actual dose delivered to the target was lower than the prescribed dose, leading to an increase risk of local recurrence. The RAT algorithm would not be recommended for SBRT of peripheral lung tumors regardless of the target size, as its dose deviations were significantly different from the MC algorithm. However, the RAT algorithm could be considered for large central lung tumors because its performance was comparable to the MC algorithm with a much shortened dose computation time.

## V. CONCLUSIONS

In the radiotherapy of NSCLC using CyberKnife, the RAT algorithm was not able predict the dose distribution as accurately as the MC algorithms. A larger difference between the two algorithms was found in the treatment of small peripheral tumors than large central tumors.
